# Impact of cryopreservation on tetramer, cytokine flow cytometry, and ELISPOT

**DOI:** 10.1186/1471-2172-6-17

**Published:** 2005-07-18

**Authors:** Holden T Maecker, James Moon, Sonny Bhatia, Smita A Ghanekar, Vernon C Maino, Janice K Payne, Kristine Kuus-Reichel, Jennie C Chang, Amanda Summers, Timothy M Clay, Michael A Morse, H Kim Lyerly, Corazon DeLaRosa, Donna P Ankerst, Mary L Disis

**Affiliations:** 1 BD Biosciences, San Jose, USA; 2 Southwest Oncology Group Statistical Center at Fred Hutchinson Cancer Research Center, Seattle, USA; 3 Beckman-Coulter, San Diego, USA; 4 Departments of Surgery, Medicine, Pathology, and Immunology, and Duke Comprehensive Cancer Center, Duke University Medical Center, Durham, USA; 5 Tumor Vaccine Group, Division of Oncology, University of Washington, Seattle, USA

## Abstract

**Background:**

Cryopreservation of PBMC and/or overnight shipping of samples are required for many clinical trials, despite their potentially adverse effects upon immune monitoring assays such as MHC-peptide tetramer staining, cytokine flow cytometry (CFC), and ELISPOT. In this study, we compared the performance of these assays on leukapheresed PBMC shipped overnight in medium versus cryopreserved PBMC from matched donors.

**Results:**

Using CMV pp65 peptide pool stimulation or pp65 HLA-A2 tetramer staining, there was significant correlation between shipped and cryopreserved samples for each assay (p ≤ 0.001). The differences in response magnitude between cryopreserved and shipped PBMC specimens were not significant for most antigens and assays. There was significant correlation between CFC and ELISPOT assay using pp65 peptide pool stimulation, in both shipped and cryopreserved samples (p ≤ 0.001). Strong correlation was observed between CFC (using HLA-A2-restricted pp65 peptide stimulation) and tetramer staining (p < 0.001). Roughly similar sensitivity and specificity were observed between the three assays and between shipped and cryopreserved samples for each assay.

**Conclusion:**

We conclude that all three assays show concordant results on shipped versus cryopreserved specimens, when using a peptide-based readout. The assays are also concordant with each other in pair wise comparisons using equivalent antigen systems.

## Background

An increasing number of experimental vaccines are being developed for diseases in which cellular immunity is likely to be required for protection [1]. These include HIV [2], hepatitis C [3], malaria [4], and cancer [5]. In these settings, immunological monitoring of antigen-specific T cell responses is likely to be an important part of vaccine assessment [6-10]. Unfortunately, surrogate markers of protection have not been established for vaccines that induce cellular immunity, although correlations between T cell IFNγ production and clinical responses have been reported in small numbers of patients [11,12] and in mouse models [13-17].

Even in the absence of surrogate markers of protection, the degree to which vaccines can induce T cell responses can be taken as a measure of immunogenicity, or potency, and as such can be used to compare different vaccine candidates. It is likely that inducing a particular level of antigen-specific T cell response to a vaccine will be necessary, though perhaps not sufficient, for vaccine efficacy of either prophylactic or therapeutic vaccines [9,10].

Traditional assays of cellular immunity have been bulk assays, including proliferation assays measuring ^3^H-thymidine incorporation [18] or cytotoxicity assays measuring ^51^Cr release [19]. These are increasingly being replaced by single-cell assays, such as MHC-peptide tetramer staining [20], cytokine flow cytometry (CFC) – also known as intracellular cytokine staining (ICS) [21,22], and enzyme-linked immunospot (ELISPOT) [23]. These assays tend to be more quantitative, in that they report a fraction of T cells or PBMC that bind a particular MHC-peptide combination or that produce a particular cytokine in response to an antigen.

Comparisons of tetramer, CFC, and/or ELISPOT have been published [12,24-31], but the relative effect of PBMC cryopreservation on each of these assays has not been examined in a side-by-side fashion. It is known that cryopreservation can negatively impact functional responses [32-35], particularly to nominal antigens. One effect of cryopreservation appears to be the loss of antigen processing capability, as measured by a disproportionate loss of responses to protein antigens, as compared to peptides [36]. There is also a relationship between viability post-thawing and capacity for functional responses [37].

The method of cryopreservation can have a tremendous impact upon viability and function [37-39]. Nevertheless, some authors have reported equivalent results in ELISPOT for fresh and frozen samples, when using an optimized protocol [39-42]. For the present study, an optimized cryopreservation protocol was developed (Disis et al., manuscript submitted) [see Additional file 1]. Two factors that had a positive impact upon cell viability and recovery, and which were incorporated into this optimized protocol, included: (1) the use of human serum albumin as a protein source in the freezing medium, and (2) the use of warmed medium for initial dilution of cells after thawing.

Using this optimized cryopreservation protocol, the present study was conducted in order to determine: (1) the correlation of fresh and cryopreserved results for tetramer, CFC, and ELISPOT assays; (2) the sensitivity and specificity of each assay using CMV seropositive and CMV seronegative donors; and (3) the inter-assay correlations using fresh versus optimally cryopreserved cells. Fresh and cryopreserved PBMC from leukapheresed healthy donors were overnight shipped, in blinded fashion, to three different laboratories, each of which was an experienced practitioner of one of the three assays. These laboratories reported results for their assay to a statistical core, where the results were compiled and statistical analyses carried out.

## Results

### 1. Fresh versus cryopreserved assay correlations

**Figure 1:**
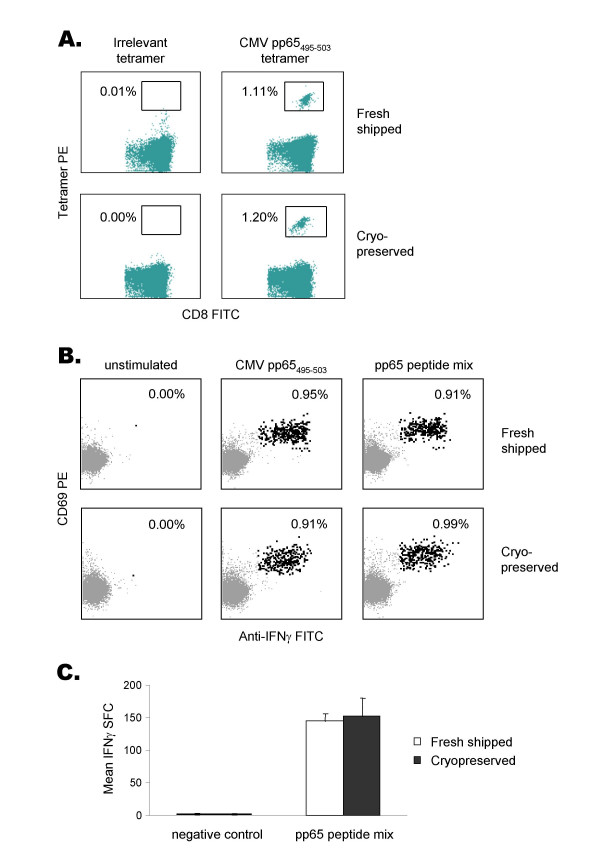
**Representative data from the three assays on the same CMV seropositive donor PBMC. **(A) Tetramer staining after gating as described in the Methods. Background seen in staining of fresh cells with negative tetramer was caused by streptavidin-PE. Frozen cells were stained with a different lot of negative tetramer. (B) CFC staining after gating as described in the Methods. (C) ELISPOT data as analyzed on the Automated Reader System as described in the Methods. Error bars represent the S.D. of 6 replicates.

**Figure 2:**
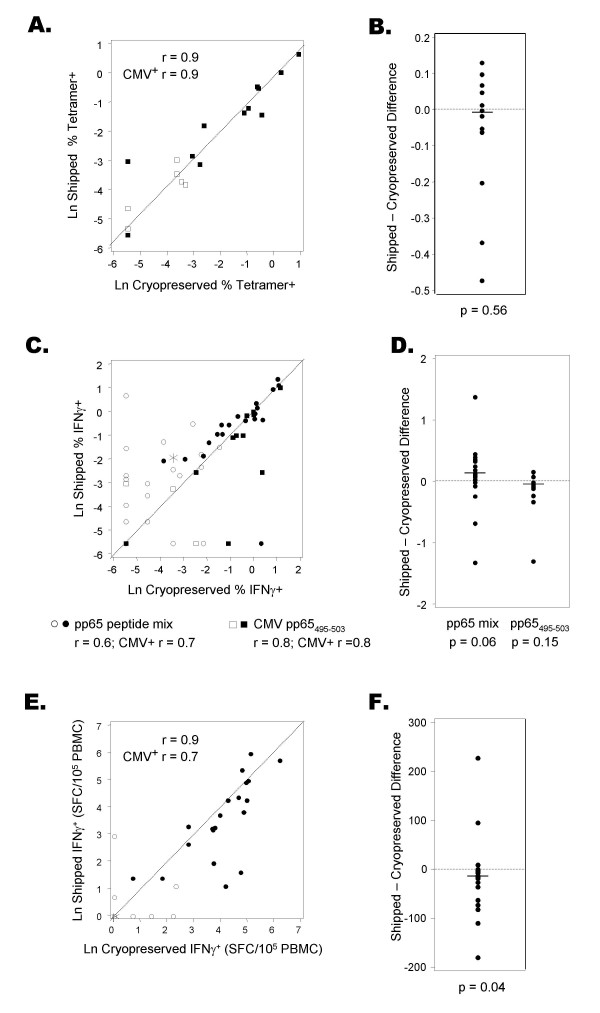
**Correlation of fresh-shipped and cryopreserved samples in all three assays. **(A, C, E) Correlation graphs of fresh-shipped versus cryopreserved PBMC samples for tetramer, CFC, and ELISPOT, respectively. Correlation coefficients (r) are shown for all data as well as for CMV seropositive donors only. Open symbols represent CMV seronegative donors; closed symbols, seropositive donors (*, CMV serostatus unknown). The diagonal line represents the line of perfect agreement between the assays. (B, D, F) Within-donor differences are shown for fresh-shipped versus cryopreserved responses. Bars represent the median difference of all donors. All statistics are based on a natural logarithm transformation, which was done to better approximate a bivariate normal distribution.

PBMC derived from leukapheresis of 20 CMV seropositive and 21 CMV seronegative donors were analyzed as fresh samples (overnight shipped) or frozen samples (cryopreserved then shipped on dry ice). Cells were processed and analyzed in a blinded fashion by tetramer staining, CFC, and ELISPOT. Representative data for each assay is shown in Figure 1. Correlations of fresh versus frozen data were performed, and are shown in Figure 2.

#### Fresh versus frozen tetramer results

20 HLA-A0201^+ ^donors (12 CMV seropositive and 8 CMV seronegative) were analyzed by tetramer staining, using an HLA-A0201 tetramer loaded with CMV pp65_495–503 _peptide. Both seropositive and seronegative donors were analyzed together to determine whether putative negative results were similar in fresh versus frozen assays, as well as putative positive results. Frequencies of tetramer^+ ^cells in fresh versus frozen samples from these donors were significantly correlated (r = 0.9, 95% C.I.: 0.9–1.0; p < 0.001) (Figure 2A). To be sure that this was not simply based upon the correlation of the negative results alone, the statistics were recalculated on CMV seropositive data only, and the correlation coefficient remained 0.9 (95% C.I.: 0.7–1.0).

To determine the bias between fresh and frozen samples from the CMV seropositive donors, the frequency of tetramer^+ ^cells in frozen samples was subtracted from that of the respective fresh samples (Figure 2B). The median difference in responses was -0.008 (95% C.I.: -0.2 to 0.07). The Wilcoxon signed-rank test was unable to detect a significant bias towards either sample type.

#### Fresh versus frozen CFC

CD8^+ ^T cell IFNγ production was measured by CFC using two different CMV antigens for stimulation: (1) a mixture of overlapping peptides corresponding to the CMV pp65 protein; and (2) the pp65_495–503 _peptide. The former stimulus was used for comparison to ELISPOT, while the latter allowed comparison to tetramer staining. CFC responses in fresh and frozen PBMC samples from all donors correlated significantly with both antigens. The estimated correlation coefficient for CMV pp65 peptide mix stimulated samples was 0.6 (95% C.I.: 0.4–0.8) (p < 0.001), and that for CMV-A2 peptide stimulated samples was 0.8, (95% C.I.: 0.6–0.8) (p < 0.001) (Figure 2C). These coefficients did not change noticeably when only CMV seropositive data points were considered (r = 0.7 (95% C.I.: 0.3–0.9) and 0.8 (95% C.I.: 0.4–0.9), respectively). Positive correlation of fresh and frozen samples was also observed for SEB-stimulated samples (r = 0.5, p = 0.002; data not shown).

To determine a bias towards a particular sample type, the difference between responses of fresh and frozen samples from CMV seropositive donors were calculated (Figure 2D). The median difference in responses to CMV-A2 peptide was -0.04 (95% C.I.: -0.22 to 0.00) and the median difference to CMV pp65 peptide mix was 0.13 (95% C.I.: 0.04–0.34). The Wilcoxon signed-rank test was unable to detect a significant bias towards either sample type stimulated with either of the two CMV antigens. However, there was a trend toward higher fresh responses to CMV pp65 peptide mix (p = 0.06).

CFC responses to pp65 peptide mix and SEB in CD4^+ ^T cells were also measured, and showed similar correlation between fresh and frozen samples (r = 0.6 and 0.4, respectively, with p < 0.001 for both stimuli) (data not shown).

#### Fresh versus frozen ELISPOT

IFNγ producing cells were measured by ELISPOT using either CMV pp65 peptide mix or SEB, and enumerated in replicates of 6 wells. The responses of fresh and frozen samples from all donors correlated significantly for pp65 peptide mix stimulation (r = 0.9, 95% C.I.: 0.8–0.9; p < 0.001) (Figure 2E). The correlation coefficient was somewhat lower when only CMV seropositive data points were considered (r = 0.7, 95% C.I.: 0.4–0.9). Interestingly, no significant correlation was observed for SEB activated samples (data not shown).

Fresh versus frozen bias was determined by subtracting pp65 peptide mix responses of frozen samples from their respective fresh samples, for CMV seropositive donors (Figure 2F). The median difference in responses was -12.5, (95% C.I.: -2 to -34). There was a significant bias toward higher responses in frozen samples (p = 0.04).

### 2. Sensitivity and specificity measurements

**Figure 3:**
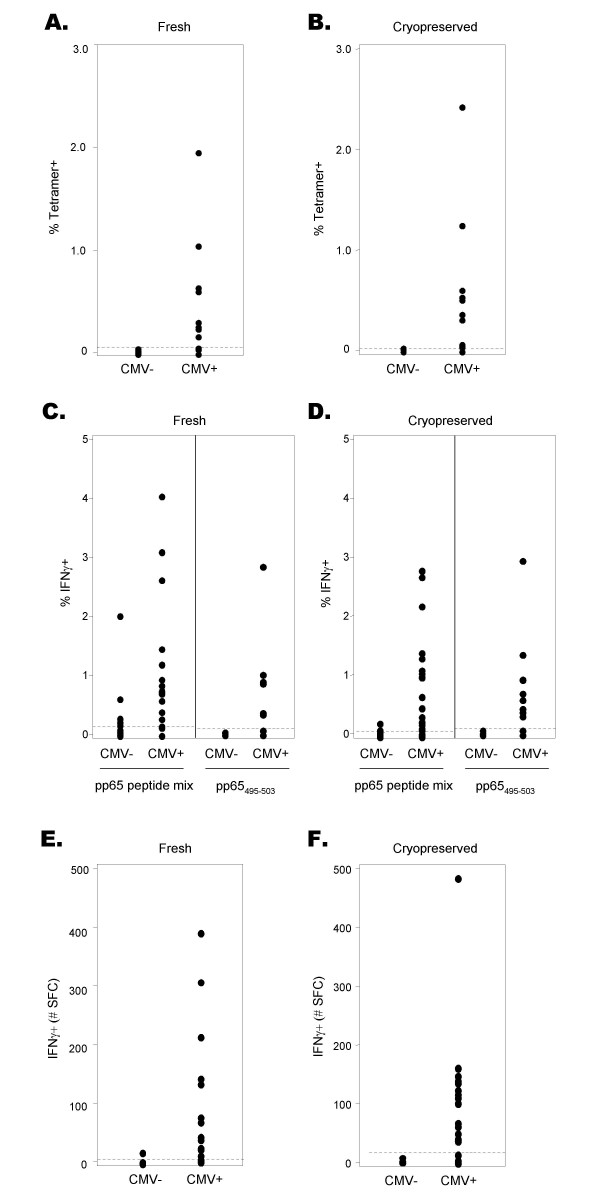
**Results of the three assays on CMV seronegative versus seropositive donors. **(A and B) Tetramer results on fresh-shipped and cryopreserved PBMC samples, respectively. (C and D) CFC results on fresh-shipped and cryopreserved PBMC samples, respectively. (E and F) ELISPOT results on fresh-shipped and cryopreserved PBMC samples, respectively. The dotted line represents the suggested cutoff based upon maximum specificity for sensitivity ≥90%, or that which obtains maximum sensitivity if maximum sensitivity <90%.

To further characterize the relative performance of the three assays, they were examined for their ability to predict CMV serostatus, using fresh and frozen PBMC. Side-by-side dot plots (Figure 3) show the results on CMV seronegative versus seropositive donors for each assay.

Sensitivity was defined as the proportion of CMV seropositive donors correctly identified using a particular cutoff, while specificity was defined as 1 – the false positive rate at that cutoff. These values vary inversely with each other, depending upon the cutoff value used to classify results as positive or negative. To quantitatively compare assay performance, the highest attainable specificity for a sensitivity of ≥90% (if achievable) was reported for each assay (Table 1).

#### Sensitivity and specificity of tetramer staining

For tetramer staining on fresh samples (Figure 3A), the median number of tetramer^+ ^cells was 0.02% for CMV seronegative donors, and 0.26% for CMV seropositive donors (n = 8 and 12, respectively). For frozen samples (Figure 3B), these medians were 0.01% and 0.26%, respectively.

A positive/negative cutoff of 0.03% for fresh samples and 0.02% for cryopreserved samples was determined as described above. Neither sample type yielded 100% sensitivity and specificity (Table 1). Additionally, there was no consistent change in sensitivity and specificity between fresh and cryopreserved data sets.

#### Sensitivity and specificity of CFC

For fresh CFC samples (Figure 3C), the median response was 0.07% for seronegative donors and 0.71% for seropositive donors (n = 20 and 21, respectively). For frozen samples (Figure 3D), these medians were 0.01% and 0.58%, respectively.

For CMV pp65 peptide mix stimulation, positive/negative cutoff values of 0.13% for fresh samples and 0.05% for frozen samples were calculated as described above. For CMV pp65_495–503 _peptide stimulation, the cutoffs were 0.08% for both fresh and frozen samples. Again, none of these assays reached 100% sensitivity and specificity (Table 1). There was also no consistent change in sensitivity and specificity between fresh and cryopreserved data sets.

#### Sensitivity and specificity of ELISPOT

For fresh ELISPOT samples (Figure 3E), the median number of SFC was 0 for seronegative donors and 34 for seropositive donors. For frozen samples (Figure 3F), these medians were 1 and 67, respectively. These numbers reiterate the bias in ELISPOT toward higher responses in frozen versus fresh samples in this study.

When positive/negative cutoff values were calculated as above, these were 4 SFC for fresh samples and 16 SFC for frozen samples. As with the other assays, 100% sensitivity and specificity was not reached (Table 1). Also, there was no consistent change in sensitivity and specificity between fresh and cryopreserved data sets.

Another way to compare sensitivity and specificity between assays and formats is to plot the sensitivity versus false positive rate in an ROC plot, and then calculate the area under this curve. The greater the area, the greater the ability of the assay to discriminate positive from negative samples. In comparing these areas (Table 1), no statistically significant differences were found between fresh and frozen samples for any of the assays, or between CFC and ELISPOT, or CFC and tetramer (all p values >0.05). This is despite the fact that tetramer and CFC results were calculated only for defined subsets of cells, whereas all PBMC were used in ELISPOT. Better discrimination might be obtained for CFC if CD4^+ ^T cell responses were also included, or for tetramer if class II and/or additional class I epitope tetramers had been used. In fact, when CFC responses for pp65 peptide mix were recalculated to include all IFNγ^+ ^PBMC, not just CD8^+ ^T cells, the area under the ROC curve was, on average, higher (0.890 for fresh, 0.915 for frozen PBMC), but still not significantly different from ELISPOT.

### 3. Inter-assay correlations

**Figure 4:**
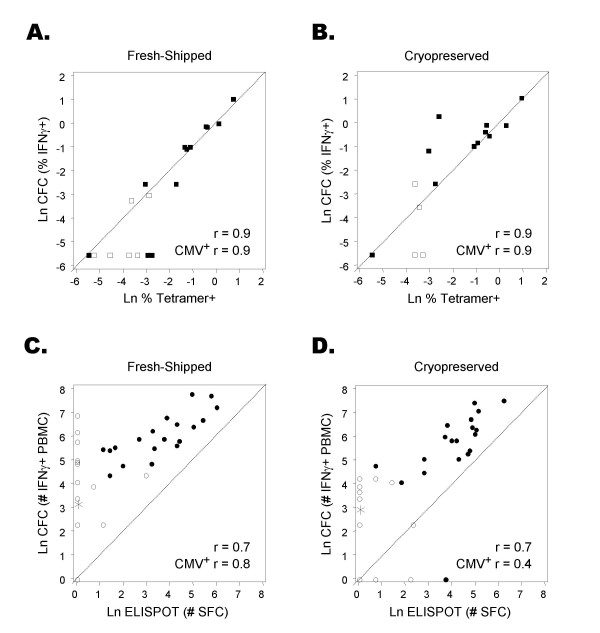
**Inter-assay correlations**. (A and B) Correlation of tetramer staining and CFC in fresh-shipped and cryopreserved PBMC samples, respectively. (C and D) Correlation of ELISPOT and CFC in fresh-shipped and cryopreserved PBMC samples, respectively, with CFC results reported as number of IFNγ^+ ^cells per 10^5 ^PBMC. In panels C-D, the mean of 6 replicates is shown for all ELISPOT data. Correlation coefficients (r) are shown for all data as well as for CMV seropositive donors only. Open symbols represent CMV seronegative donors; closed symbols, seropositive donors (*, CMV serostatus unknown). The diagonal line represents the line of perfect agreement between the assays.

Two-way correlations were performed between tetramer staining and CFC, and between CFC and ELISPOT (Figure 4). CMV pp65_495–503_-specific responses obtained using CFC were compared to HLA-A0201 pp65_495–503 _tetramer staining for all 20 HLA-A0201^+ ^donors in the study (12 CMV seropositive, 8 CMV seronegative). CMV pp65 peptide mix-specific responses obtained by CFC and ELISPOT were compared for all 20 CMV seropositive and 21 CMV seronegative donors in the study.

#### Tetramer staining versus CFC

These assays correlated significantly with each other for both fresh and frozen samples (Figures 4A and 4B). The estimated correlation coefficient was 0.9 for both fresh and frozen samples (95% C.I.: 0.8–1.0 for both) (p < 0.001 for both). These were the strongest inter-assay correlations observed in this study.

#### CFC versus ELISPOT

A significant correlation between these two assays was observed using both fresh and frozen PBMC samples (data not shown). The estimated correlation coefficient was 0.5 (95% C.I.: 0.2–0.7) (p = 0.001) for fresh samples, and 0.7 (95% C.I.: 0.5–0.8) (p < 0.001) for frozen samples. However, when only CMV seropositive donors were considered, the p values became non-significant for both fresh and frozen samples. Thus, CFC and ELISPOT were less tightly correlated than tetramer and CFC. This is in agreement with another published report comparing ELISPOT and CFC [29].

The scales and readouts of CFC and ELISPOT are very different (%IFNγ^+ ^CD8^+ ^T cells versus SFC per 10^5 ^PBMC). To correct for these differences, the CFC results were expressed as the total number of IFNγ^+ ^cells per 10^5 ^PBMC, without gating on CD3 or CD8. This mimicked the readout of the ELISPOT assay, which reported positive events per 10^5 ^PBMC (the number of cells plated in each ELISPOT well). The estimated correlation coefficients under these conditions were 0.7 for both fresh (95% CI: 0.4–0.8) and frozen (95% CI: 0.5–0.8) samples (p < 0.001 both) (Figure 4C and 4D). When only CMV seropositive samples were considered, the correlation coefficients became 0.8 (95% CI: 0.5–0.9) and 0.4 (95% CI: 0.0–0.7), respectively (p < 0.001 and p = 0.06, respectively). Thus, expression of the CFC results in this format appeared to improve the correlation. This may reflect the response of non-CD8^+ ^cells in these assays. Note also that when CFC results were plotted in this way, there were on average several-fold higher responses with CFC versus ELISPOT (as represented by most points being above the diagonal in Figures 4C and 4D).

## Discussion

This study represents a comprehensive analysis of the effect of cryopreservation (using an optimized cryopreservation protocol) on tetramer, CFC, and ELISPOT assays using peptide-based antigens. Each assay was individually optimized by a laboratory experienced in that technique, to ensure the best possible performance for each. For example, costimulatory antibodies (CD28 and CD49d) were used in CFC but not ELISPOT, despite the fact that addition of CD28 antibody can improve ELISPOT sensitivity [43]. In our hands, occasional donors developed unacceptably high ELISPOT backgrounds (>100 spots per 10^6 ^PBMC) with the use of CD28 costimulation (data not shown), so it was not used for that assay, although it was used for CFC. While there are shortcomings of this study design, we felt that this provided the fairest and most robust way to compare these assays. The assays were compared by correlation of results for fresh and frozen samples; by analyzing sensitivity and specificity of each assay on fresh and frozen samples; and by determining inter-assay correlations using fresh and frozen samples. The overall findings are summarized in Table 2.

None of the three assays showed a significant reduction of signal in frozen cells relative to fresh cells. The fresh-to-frozen correlation was strongest for tetramer staining, which does not rely on cell function, then CFC and ELISPOT. Compared to CMV responses, SEB responses were less well correlated in fresh and frozen samples using CFC, and not at all correlated using ELISPOT. The reasons for this are not clear; however, they may stem from the relative affinities of T cells responding to CMV peptides versus SEB. It is known that T cells bearing a number of different TCR Vβ sequences can participate, to varying degrees, in the SEB response. The participation of different Vβ families is related to their affinity for SEB. Low affinity Vβ responses may be preferentially lost upon cryopreservation. The differential representation of these Vβ subsets in different donors may thus lead to inconsistencies in the correlation of fresh to cryopreserved responses for SEB. CMV responses may be of generally uniform and higher affinity, as suggested by their typically bright, clustered IFNγ staining (compared to SEB responses, where IFNγ staining tends to be more of a smear). If this is true, it may also suggest that other low-affinity responses, such as those to tumor antigens, may be more susceptible to loss upon cryopreservation; this needs to be tested.

Rather than compare cryopreserved PBMC to fresh, same-day activated PBMC, the former were compared to fresh, overnight-shipped PBMC from leukapheresed donors. Since some functional degradation undoubtedly occurs with overnight shipping, this is not ideal in terms of assessing the total signal loss due to cryopreservation. However, the reality of large clinical studies is that PBMC will in all likelihood be cryopreserved and/or shipped overnight to a laboratory that does immunological monitoring. Thus, comparison of these two conditions represents a comparison of two likely scenarios for handling of PBMC samples in clinical trials. The current results imply that there is unlikely to be a pronounced difference in results with any of the three assays when using either of these conditions. It is unknown whether a detectable loss of signal would be observed if PBMC were subjected to both overnight shipping and then cryopreservation. Of course, all of these data assume that reasonable care is taken in sample cryopreservation and shipping, which were optimized for this study (Disis et al., manuscript submitted). Also, the stability of healthy donor cells, as used in this study, may be superior to those from certain disease states, e.g., HIV or tumor patients. Finally, the use of shipped PBMC (rather than whole blood) may have improved the results seen in this study; thus our results should not be taken as indicative of what would be achieved if whole blood samples were shipped overnight.

Because this study used peptide-based antigens (and SEB), drastic loss of functional responses as reported for whole-protein antigens [36] were not seen. The current results should not be interpreted to apply to non-peptide antigens, since antigen-processing capabilities are preferentially lost upon cryopreservation.

We defined sensitivity and specificity on the basis of reactivity with donors who were CMV seropositive, and lack of reactivity with donors who were CMV seronegative. This comparison is not ideal, since there are reports of non-correlation of serological and T cell responses to CMV [44]. In particular, assays examining a single epitope response (e.g., tetramer and CFC for pp65_495–503_) may underestimate CMV responders, due to immunodominance hierarchies [45]. HLA-A2-restricted responses to pp65_495–503 _are known to be suppressed in individuals co-expressing HLA-B7 [46], and such individuals were not excluded from this study. However, CFC assays using pp65 peptide mix were found in at least one previous study to differentiate CMV seropositive and seronegative donors with 100% sensitivity and specificity, although the sample size was small (15 seropositive and 14 seronegative donors) [47]. All three assays were recently compared in a study of fresh PBMC from 21 CMV seropositive and 20 CMV seronegative healthy donors, with a sensitivity of 87.5% and specificity of 100% for each assay [48].

None of the three assays attained 100% sensitivity and specificity using either fresh or cryopreserved PBMC in the present study. ELISPOT, especially on cryopreserved PBMC, showed slightly greater sensitivity (for specificity ≥90%) than did CFC or tetramer (although the difference was not statistically significant). This was partly due to the reactivity of one or two seronegative donors in the CFC and tetramer assays. This reactivity was more pronounced in the assays with fresh PBMC, but was still present in the cryopreserved PBMC from the same donors. In the case of CFC, the cryopreserved PBMC assay was repeated on additional cells and still showed a similar level of reactivity, suggesting that it was not due to a technical error. It is possible that these donors represented true discordant responses between serological and cellular assays.

The quantitative comparison of tetramer and CFC resulted in the tightest correlation. This could be related to the fact that both of these assays use the same readout platform (flow cytometry). The correlation of CFC and ELISPOT was less precise, and was not statistically significant when CMV seronegative donors were excluded. The correlation appeared tighter when CFC results were expressed as a proportion of all PBMC. This could be due to variable proportions of CD4^+ ^T cells contributing to the response to pp65 peptide mix. It is also possible that non-T cells contribute to this response, although this was not directly assessed in this study.

## Conclusion

We conclude that tetramer, CFC, and ELISPOT assays can be performed on optimally cryopreserved PBMC with minimal or no loss of signal when compared to fresh, overnight-shipped PBMC. The assays correlate significantly in direct comparisons using the same antigen systems, whether fresh or cryopreserved PBMC are used. The strongest correlations of fresh and cryopreserved PBMC are seen with tetramer and CFC assays; and these two assays also correlate most strongly with each other. All three assays showed roughly similar sensitivity and specificity in discriminating CMV seropositive from seronegative donors. The strong correlation of tetramer and CFC assays in fresh and cryopreserved cells, along with their multiparameter information content, make them ideal choices for immune monitoring assays.

## Methods

### PBMC isolation and processing

PBMC from leukapheresis (obtained from healthy donors without cytokine mobilization) were isolated using Ficoll gradient separation. Briefly, 5 ml of leukapheresis product were aliquoted into 50 ml conical tubes (BD Falcon, Franklin Lakes, NJ) washed once by adding HBSS (Gibco Invitrogen Corporation, Grand Island, NY) and centrifuged for 10 minutes at 280 × G. The pelleted cells were resuspended and 40 ml of HBSS were added. Ten ml of Ficoll Paque (Amersham Biosciences, Piscataway, NJ) were carefully underlayed and the tubes centrifuged at 400 × G for 40 minutes. The buffy coat was collected and washed twice with HBSS. Viability was assessed using 0.4% Trypan blue (Sigma, St. Louis, MO). For fresh-shipped specimens, 2 × 10^7 ^viable lymphocytes were resuspended in 50 ml RPMI+10% fetal bovine serum and shipped overnight at ambient temperature in a 50 ml conical tube packed in an insulated foam container. Fresh-shipped PBMC were centrifuged as soon as received and the assays set up as described below.

### Cryopreservation [see Additional file 1]

To cryopreserve PBMC, 2X freezing media was first prepared, containing 20% DMSO in RPMI (Sigma Chemical Co., St. Louis, MO) containing 12.5% human serum albumin (HSA) (Gemini Bioproducts, Woodland, CA), and cooled on ice for a minimum of 30 minutes. Ficolled PBMC at 2 × 10^7 ^viable lymphocytes/ml were resuspended in cooled RPMI+12.5% HSA with no DMSO. An equal volume of chilled 2X freezing media was added to the cell suspension dropwise, while gently swirling the tube. One ml of this cell suspension was aliquoted into each cryovial (Sarstedt, Inc., Newton, NC). Once aliquoted, cryovials were placed on ice and then transferred into a freezing container (Nalgene, Rochester, NY), and stored at -80°C for 24 hours. Cryovials were then transferred into liquid nitrogen for long-term storage. After 30 days, cryovials were overnight shipped on dry ice to the recipient laboratories.

### Thawing [see Additional file 1]

Cryopreserved PBMC were stored at -80°C until thawing to set up the assays. Cryopreserved cells were thawed and slowly diluted with 8 ml of warm RPMI+10% fetal bovine serum+antibiotics (cRPMI-10, all components from Sigma). The cells were centrifuged for 7 minutes at 250 × G, then resuspended as described below for each assay. Viability and recovery were checked using Trypan blue, and were >80% and >50%, respectively, in all samples.

### MHC class I-peptide tetramer staining

Fresh and frozen PBMC from 12 CMV seropositive and 8 CMV seronegative patients were screened with MHC tetramer composed of HLA-A*0201 monomers carrying the CMV pp65_495–503 _peptide epitope (NLVPMVATV). For flow cytometry analysis, the Multiple Antibody Single Color protocol (iMASC, Beckman Coulter Inc., Fullerton, CA) was used. Briefly, ten μl each of CD4, CD13, and CD19 antibodies conjugated to PE-Cy5 (PC5) were added to sample tubes in order to exclude CD4 T cells, granulocytes, and B cells from analysis. In addition, 10 μl of CD8 FITC and 10 μl tetramer PE were added, followed by 1 × 10^6 ^PBMC in 100 μl of flow cytometry buffer (HBSS containing 0.1% bovine serum albumin, 0.02% sodium azide). Samples were incubated for 30 minutes at room temperature followed by a wash with flow cytometry buffer and fixation in 1% formaldehyde. Samples were run on a BD FACSCalibur flow cytometer that was set to acquire 30,000 CD8^+ ^T cells. Analysis was performed using CellQuest software (BD Biosciences, San Jose, CA) and gating was done to accept CD8^+ ^/tetramer^+ ^cells and to exclude PC5-positive cells.

### Cytokine flow cytometry

CFC assays were performed according to a previously published method [49,50]. 200 μl containing 2 × 10^6 ^PBMC in cRPMI-10 medium were plated per well in 96-well round-bottom plates. For cryopreserved PBMC, the thawed cells were then rested at 37°C, 7% CO_2 _overnight. For both fresh and cryopreserved PBMC, activation reagents (stimulus + brefeldin A) were added in a volume of 20 μl per 200 μl of cell suspension per well and the cells were then incubated at 37°C for 6 hours. Stimuli included CMV pp65 peptide mix (BD Biosciences; used at a final concentration of 1.7 μg/ml/peptide); CMV pp65_495–503 _peptide (SynPep Corp., Dublin, CA; used at a final concentration of 10 μg/ml); and SEB (Sigma; used at a final concentration of 1 μg/ml). All samples received a final concentration of 1 μg/ml each of CD28+CD49d costimulatory antibodies and 10 μg/ml of brefeldin A (both from BD Biosciences). After 6 hours incubation, the cells were treated with 2 mM final concentration of EDTA for 15 minutes at room temperature, then fixed with FACS Lysing Solution (BD Biosciences) and stored at -80°C. When ready to stain, the frozen plates were thawed at 37°C and processed further with FACS Permeabilizing Solution 2 (BD Biosciences) followed by staining with IFNγ FITC/CD69 PE/CD8 PerCPCy5.5/CD3 APC (BD Biosciences) for 1 hour at room temperature. Plates were washed and cells resuspended in 1% paraformaldehyde in PBS.

Samples were acquired within 24 hours of staining using a FACSCalibur flow cytometer with a Multiwell Autosampler, using Multiwell Plate Manager and CellQuest Pro software (BD Biosciences). 40,000 CD3^+^CD8^+ ^lymphocytes were collected per sample. A "response region" was set around double-positive cells in a gated dot plot displaying CD69 versus IFNγ staining from an SEB-stimulated sample. This response region was then applied to all samples to determine the percentage of cytokine-positive cells. Data were reported as the net percent of CD3^+^CD8^+ ^lymphocytes that were IFNγ^+ ^after subtracting the response of unstimulated samples.

### ELISPOT

PBMC were assayed for IFNγ production in the presence of CMV pp65 peptide mix (BD Biosciences), SEB, and media in replicates of 6. Multiscreen-HA 96-well plates (Millipore, Bedford, MA) were coated overnight at 4°C with 100 μl/well of 10 μg/ml mouse anti-human IFNγ mAb 7-D1K (diaPharma Group, Inc., West Chester, OH) in Dulbecco's Phosphate Buffered Saline (DPBS) (Gibco Invitrogen). The plates were washed 3 times for 5 minutes each with 150 μl DPBS/well and blocked with 150 μl/well of RPMI-1640, 10% human AB serum, 25 mM HEPES, 100 U/ml penicillin, 100 μg/ml streptomycin, and 2 mM L-glutamine for 1 hour at 37°C in 5% CO_2_. PBMC were plated at 100,000 per well with 1:800 of CMV pp65 peptide mix (approximately 1.75 μg/ml of each peptide), 100 ng/ml of SEB, or media in a total volume of 200 μl/well for 18–24 hours at 37°C in 5% CO_2_.

The plates were washed with 0.05% Tween/DPBS using a Tecan 96PW plate washer (Tecan, Research Triangle Park, NC). A solution of 100 μl of mouse anti-human IFNγ biotinylated mAb 7-B6-1 (diaPharma) at 1 μg/ml in DPBS was added to each well and the plates incubated for 2 hours at 37°C, 5% CO_2_. Vectastain ABC Peroxidase (Vector Labs, Inc., Burlingame, CA) was added at 100 μl/well for 1 hour at room temperature after washing with 0.05% Tween/DPBS using the Tecan plate washer. The plates were washed for the last time with 0.05% Tween/DPBS followed by DPBS. Color was developed using 100 μl/well of 3-amino-9-ethyl-carbazole [AEC] (Sigma) reconstituted in an acetate buffer for 4 minutes at room temperature in the dark. Color development was stopped with deionized water. Basins were removed and the membranes dried overnight in the dark. Membranes were attached to sealing tape (Millipore, Bedford, MA) and the number of spots per well was determined using a KS ELISPOT Automated Reader System with KS ELISPOT 4.2 Software (Carl Zeiss, Inc., Thornwood, NY). The mean number of spots from the six replicate wells at each dilution was reported for each antigen. The analyses in this paper were based on the wells containing 1 × 10^5 ^responder PBMC, which is the dilution that yielded the highest ratio of spots/PBMC (data not shown).

### Statistical analyses

All samples tested were included in the analysis, as no attempt was made to exclude outliers. Tests of correlations between fresh and frozen samples were performed using the Pearson Product Moment Correlation Coefficient on the natural logarithm of responses. This transformation was performed to correct for observed skewness in the data, since the statistical test assumes a bivariate normal distribution. However, the significance of the correlations was largely unchanged when untransformed data was used. If the net response equaled zero, a small constant was added (0.004 for % positive and 1 for cells/10^5 ^PBMC) prior to computing the natural log. The Wilcoxon Signed-Rank test was performed for differences between paired fresh and frozen samples. Tests of correlations between assays were performed using the Pearson Product Moment Correlation Coefficient after natural logarithmic transformation and adjustment for zeroes.

For each assay and antigen combination, the operating characteristics were summarized in terms of the sensitivity and false positive rate (1-specificity) for cut-off values of net response of both fresh and frozen samples. The sensitivity and false positive rate were defined as the proportion of correctly identified CMV seropositive samples and incorrectly identified seronegative samples, respectively, with an assay response exceeding each cut-off value. The ROC curve was constructed as a plot of the false positive test rate versus sensitivity for all cut-off values in the range of assay responses observed. For each ROC curve, the sensitivity and specificity was reported for the cut-off that maximized specificity subject to the constraint that sensitivity ≥90%; or alternatively, if the sensitivity was bounded below 90%, at the specificity corresponding to the maximum sensitivity. Tests for differences in the areas under the ROC curves were performed using the nonparametric test for correlated data of Delong et al [51]. The ROC analysis was performed with Stata version 7 software (StataCorp LP, College Station, TX). All other statistical analyses were performed with SAS version 9 software (SAS Institute, Cary, NC).

## Authors' contributions

HTM wrote the manuscript with input from SAG, DPA, JM, TMC, and TKR. JM and DPA compiled the data and did statistical analysis. SB, JKP, AS, JCC, and CD processed and analyzed the samples. HTM, SAG, VCM, TMC, MAM, HKL, TKR, and MLD planned and supervised the study and all authors reviewed and edited the manuscript

## Supplementary Material

Additional File 1 Protocol for Isolation, Cryopreservation, and Thawing of PBMC Optimized protocol used in this study for cryopreservation and thawing of PBMC Click here for file

## Figures and Tables

**Table 1:** Cutoffs, sensitivity, and specificity from ROC curves

Assay	Antigen	Sample Type	Cutoff Point^1^	Sensitivity	Specificity	Area Under ROC Curve^2^
Tetramer (% +)	CMV pp65 A2 peptide	Fresh	0.05	92%	88%	0.906+/-0.076
		Cryo.	0.02	83%	100%	0.875+/-0.086

	CMV pp65 peptide mix	Fresh	0.13	90%	71%	0.819+/-0.075
CFC (% +)		Cryo.	0.05	90%	76%	0.920+/-0.046
	CMV pp65 A2 peptide	Fresh	0.08	75%	100%	0.844+/-0.084
		Cryo.	0.08	83%	88%	0.891+/-0.074

ELISPOT (# SFC)	CMV pp65 peptide mix	Fresh	4	95%	94%	0.982+/-0.018
		Cryo.	16	90%	100%	0.985+/-0.015

^1^Cut-off is that which achieves maximum specificity for sensitivity ≥90%, or that which obtains maximum sensitivity if maximum sensitivity <90%.)

^2^Values are given +/- standard error.

**Table 2:** Summary of assay characteristics

Assay	Type of assay	Readout	Fresh to frozen correlation	Sensitivity and specificity^1^	Inter-assay correlations
Tetramer	Phenotypic	4-color flow cytometry	r = 0.9	>70%^2^	r = 0.9
CFC	Functional	4-color flow cytometry	r = 0.6 (CMV) r = 0.4 (SEB)	>70%^2^	r = 0.9 r = 0.7
ELISPOT	Functional	Plate reader	r = 0.6 (CMV) NS (SEB)	>90%	r = 0.7

^1^For both fresh and frozen samples with all peptide-based stimuli.

^2^Potentially higher if multiple tetramers or CD4 and CD8 responses are considered.

## References

[B1] Zinkernagel RM, Hengartner H (2004). On immunity against infections and vaccines: credo 2004. Scand J Immunol.

[B2] McMichael AJ, Hanke T (2003). HIV vaccines 1983-2003. Nat Med.

[B3] Torresi J, Bharadwaj M, Jackson DC, Gowans EJ (2004). Neutralising antibody, CTL and dendritic cell responses to hepatitis C virus: a preventative vaccine strategy. Curr Drug Targets.

[B4] Moorthy VS, Good MF, Hill AV (2004). Malaria vaccine developments. Lancet.

[B5] Gilboa E (2004). The promise of cancer vaccines. Nat Rev Cancer.

[B6] Disis ML, Maecker HT, Clay TM, Lyerly HK, Chang JCC, Lotze MT, Thompson AW (2003). Immunologic monitoring to assess immunity to solid tumors. Measuring immunity: the immunoloic surrogates handbook.

[B7] Keilholz U, Weber J, Finke JH, Gabrilovich DI, Kast WM, Disis ML, Kirkwood JM, Scheibenbogen C, Schlom J, Maino VC, Lyerly HK, Lee PP, Storkus W, Marincola F, Worobec A, Atkins MB (2002). Immunologic monitoring of cancer vaccine therapy: results of a workshop sponsored by the Society for Biological Therapy. J Immunother.

[B8] Coulie PG, van der Bruggen P (2003). T-cell responses of vaccinated cancer patients. Curr Opin Immunol.

[B9] Maecker HT, Disis ML (2005). The role of immune monitoring in evaluating cancer immunotherapy. Immunotherapy of Cancer.

[B10] Maecker HT, Maino VC (2003). T cell immunity to HIV:  defining parameters of protection. Current HIV Research.

[B11] Belli F, Testori A, Rivoltini L, Maio M, Andreola G, Sertoli MR, Gallino G, Piris A, Cattelan A, Lazzari I, Carrabba M, Scita G, Santantonio C, Pilla L, Tragni G, Lombardo C, Arienti F, Marchiano A, Queirolo P, Bertolini F, Cova A, Lamaj E, Ascani L, Camerini R, Corsi M, Cascinelli N, Lewis JJ, Srivastava P, Parmiani G (2002). Vaccination of metastatic melanoma patients with autologous tumor-derived heat shock protein gp96-peptide complexes: clinical and immunologic findings. J Clin Oncol.

[B12] Sun P, Schwenk R, White K, Stoute JA, Cohen J, Ballou WR, Voss G, Kester KE, Heppner DG, Krzych U (2003). Protective immunity induced with malaria vaccine, RTS,S, is linked to Plasmodium falciparum circumsporozoite protein-specific CD4(+) and CD8(+) T cells producing IFN-gamma. J Immunol.

[B13] Perez-Diez A, Spiess PJ, Restifo NP, Matzinger P, Marincola FM (2002). Intensity of the vaccine-elicited immune response determines tumor clearance. J Immunol.

[B14] Gurunathan S, Prussin C, Sacks DL, Seder RA (1998). Vaccine requirements for sustained cellular immunity to an intracellular parasitic infection. Nat Med.

[B15] Gurunathan S, Stobie L, Prussin C, Sacks DL, Glaichenhaus N, Fowell DJ, Locksley RM, Chang JT, Wu CY, Seder RA (2000). Requirements for the maintenance of Th1 immunity in vivo following DNA vaccination: a potential immunoregulatory role for CD8+ T cells. J Immunol.

[B16] Stobie L, Gurunathan S, Prussin C, Sacks DL, Glaichenhaus N, Wu CY, Seder RA (2000). The role of antigen and IL-12 in sustaining Th1 memory cells in vivo: IL-12 is required to maintain memory/effector Th1 cells sufficient to mediate protection to an infectious parasite challenge. Proc Natl Acad Sci U S A.

[B17] Mendez S, Gurunathan S, Kamhawi S, Belkaid Y, Moga MA, Skeiky YA, Campos-Neto A, Reed S, Seder RA, Sacks D (2001). The potency and durability of DNA- and protein-based vaccines against Leishmania major evaluated using low-dose, intradermal challenge. J Immunol.

[B18] Gehrz RC, Knorr SO (1979). Characterization of the role of mononuclear cell subpopulations in the in vitro lymphocyte proliferation assay. Clin Exp Immunol.

[B19] McCoy JL, Herberman RB, Rosenberg EB, Donnelly FC, Levine PH, Alford C (1973). 51 Chromium-release assay for cell-mediated cytotoxicity of human leukemia and lymphoid tissue-culture cells. Natl Cancer Inst Monogr.

[B20] Altman JD, Moss PAH, Goulder PJR, Barouch DH, McHeyzer-Williams MG, Bell JI, McMichael AJ, Davis MM (1996). Phenotypic analysis of antigen-specific T lymphocytes. Science.

[B21] Waldrop SL, Pitcher CJ, Peterson DM, Maino VC, Picker LJ (1997). Determination of antigen-specific memory/effector CD4+ T cell frequencies by flow cytometry: evidence for a novel, antigen-specific homeostatic mechanism in HIV-associated immunodeficiency. J Clin Invest.

[B22] Suni MA, Picker LJ, Maino VC (1998). Detection of antigen-specific T cell cytokine expression in whole blood by flow cytometry. J Immunol Methods.

[B23] Czerkinsky C, Andersson G, Ekre HP, Nilsson LA, Klareskog L, Ouchterlony O (1988). Reverse ELISPOT assay for clonal analysis of cytokine production. I. Enumeration of gamma-interferon-secreting cells. J Immunol Methods.

[B24] Kuzushima K, Hoshino Y, Fujii K, Yokoyama N, Fujita M, Kiyono T, Kimura H, Morishima T, Morishima Y, Tsurumi T (1999). Rapid determination of Epstein-Barr virus-specific CD8(+) T-cell frequencies by flow cytometry. Blood.

[B25] Moretto WJ, Drohan LA, Nixon DF (2000). Rapid quantification of SIV-specific CD8 T cell responses with recombinant vaccinia virus ELISPOT or cytokine flow cytometry. Aids.

[B26] Asemissen AM, Nagorsen D, Keilholz U, Letsch A, Schmittel A, Thiel E, Scheibenbogen C (2001). Flow cytometric determination of intracellular or secreted IFNgamma for the quantification of antigen reactive T cells. J Immunol Methods.

[B27] Pahar B, Li J, Rourke T, Miller CJ, McChesney MB (2003). Detection of antigen-specific T cell interferon gamma expression by ELISPOT and cytokine flow cytometry assays in rhesus macaques. J Immunol Methods.

[B28] Whiteside TL, Zhao Y, Tsukishiro T, Elder EM, Gooding W, Baar J (2003). Enzyme-linked immunospot, cytokine flow cytometry, and tetramers in the detection of T-cell responses to a dendritic cell-based multipeptide vaccine in patients with melanoma. Clin Cancer Res.

[B29] Karlsson AC, Martin JN, Younger SR, Bredt BM, Epling L, Ronquillo R, Varma A, Deeks SG, McCune JM, Nixon DF, Sinclair E (2003). Comparison of the ELISPOT and cytokine flow cytometry assays for the enumeration of antigen-specific T cells. J Immunol Methods.

[B30] Heeger PS, Greenspan NS, Kuhlenschmidt S, Dejelo C, Hricik DE, Schulak JA, Tary-Lehmann M (1999). Pretransplant frequency of donor-specific, IFN-gamma-producing lymphocytes is a manifestation of immunologic memory and correlates with the risk of posttransplant rejection episodes. J Immunol.

[B31] Helms T, Boehm BO, Asaad RJ, Trezza RP, Lehmann PV, Tary-Lehmann M (2000). Direct visualization of cytokine-producing recall antigen-specific CD4 memory T cells in healthy individuals and HIV patients. J Immunol.

[B32] Oldham RK, Dean JH, Cannon GB, Ortaldo JR, Dunston G, Applebaum F, McCoy JL, Djeu J, Herberman RB (1976). Cryopreservation of human lymphocyte function as measured by in vitro assays. Int J Cancer.

[B33] Weinberg A, Wohl DA, Brown DG, Pott GB, Zhang L, Ray MG, van der Horst C (2000). Effect of cryopreservation on measurement of cytomegalovirus-specific cellular immune responses in HIV-infected patients. J Acquir Immune Defic Syndr.

[B34] Costantini A, Mancini S, Giuliodoro S, Butini L, Regnery CM, Silvestri G, Montroni M (2003). Effects of cryopreservation on lymphocyte immunophenotype and function. J Immunol Methods.

[B35] Weinberg A, Betensky RA, Zhang L, Ray G (1998). Effect of shipment, storage, anticoagulant, and cell separation on lymphocyte proliferation assays for human immunodeficiency virus-infected patients. Clin Diagn Lab Immunol.

[B36] Maecker HT, Dunn HS, Suni MA, Khatamzas E, Pitcher CJ, Bunde T, Persaud N, Trigona W, Fu TM, Sinclair E, Bredt BM, McCune JM, Maino VC, Kern F, Picker LJ (2001). Use of overlapping peptide mixtures as antigens for cytokine flow cytometry. J Immunol Methods.

[B37] Weinberg A, Zhang L, Brown D, Erice A, Polsky B, Hirsch MS, Owens S, Lamb K (2000). Viability and functional activity of cryopreserved mononuclear cells. Clin Diagn Lab Immunol.

[B38] Kleeberger CA, Lyles RH, Margolick JB, Rinaldo CR, Phair JP, Giorgi JV (1999). Viability and recovery of peripheral blood mononuclear cells cryopreserved for up to 12 years in a multicenter study. Clin Diagn Lab Immunol.

[B39] Kreher CR, Dittrich MT, Guerkov R, Boehm BO, Tary-Lehmann M (2003). CD4+ and CD8+ cells in cryopreserved human PBMC maintain full functionality in cytokine ELISPOT assays. J Immunol Methods.

[B40] Smith JG, Liu X, Kaufhold RM, Clair J, Caulfield MJ (2001). Development and validation of a gamma interferon ELISPOT assay for quantitation of cellular immune responses to varicella-zoster virus. Clin Diagn Lab Immunol.

[B41] Gebauer BS, Hricik DE, Atallah A, Bryan K, Riley J, Tary-Lehmann M, Greenspan NS, Dejelo C, Boehm BO, Hering BJ, Heeger PS (2002). Evolution of the enzyme-linked immunosorbent spot assay for post-transplant alloreactivity as a potentially useful immune monitoring tool. Am J Transplant.

[B42] Sobota V, Bubenik J, Indrova M, Vlk V, Jakoubkova J (1997). Use of cryopreserved lymphocytes for assessment of the immunological effects of interferon therapy in renal cell carcinoma patients. J Immunol Methods.

[B43] Ott PA, Berner BR, Herzog BA, Guerkov R, Yonkers NL, Durinovic-Bello I, Tary-Lehmann M, Lehmann PV, Anthony DD (2004). CD28 costimulation enhances the sensitivity of the ELISPOT assay for detection of antigen-specific memory effector CD4 and CD8 cell populations in human diseases. J Immunol Methods.

[B44] Zhu J, Shearer GM, Marincola FM, Norman JE, Rott D, Zou JP, Epstein SE (2001). Discordant cellular and humoral immune responses to cytomegalovirus infection in healthy blood donors: existence of a Th1-type dominant response. Int Immunol.

[B45] Betts MR, Casazza JP, Patterson BA, Waldrop S, Trigona W, Fu TM, Kern F, Picker LJ, Koup RA (2000). Putative immunodominant human immunodeficiency virus-specific CD8(+) T- cell responses cannot be predicted by major histocompatibility complex class I haplotype. J Virol.

[B46] Ghanekar SA, Nomura LE, Suni MA, Picker LJ, Maecker HT, Maino VC (2001). Gamma interferon expression in CD8(+) T cells is a marker for circulating cytotoxic T lymphocytes that recognize an HLA A2-restricted epitope of human cytomegalovirus phosphoprotein pp65. Clin Diagn Lab Immunol.

[B47] Dunn HS, Haney DJ, Ghanekar SA, Stepick-Biek P, Lewis DB, Maecker HT (2002). Dynamics of CD4 and CD8 T cell responses to cytomegalovirus in healthy human donors. J Infect Dis.

[B48] Hobeika AC, Morse MA, Osada T, Ghanayem M, Niedzwiecki D, Barrier R, Lyerly HK, Clay TM (2005). Enumerating antigen-specific T-cell responses in peripheral blood: a comparison of peptide MHC Tetramer, ELISpot, and intracellular cytokine analysis. J Immunother.

[B49] Suni MA, Dunn HS, Orr PL, deLaat R, Sinclair E, Ghanekar SA, Bredt BM, Dunne JF, Maino VC, Maecker HT (2003). Performance of plate-based cytokine flow cytometry with automated data analysis. BMC Immunology.

[B50] Maecker HT, Hawley TS, Hawley RG (2004). Cytokine flow cytometry. Flow Cytometry Protocols.

[B51] DeLong ER, DeLong DM, Clarke-Pearson DL (1988). Comparing the areas under two or more correlated receiver operating characteristic curves: a nonparametric approach. Biometrics.

