# Supplementary material for: Impact of cryopreservation on tetramer, cytokine flow cytometry, and ELISPOT
Source: BMC Immunol. 2005 Jul 18;6:17. doi: 10.1186/1471-2172-6-17 (PMC1190174; doi:10.1186/1471-2172-6-17)
Supplement: Additional File 1 — Protocol for Isolation, Cryopreservation, and Thawing of PBMC Optimized protocol used in this study for cryopreservation and thawing of PBMC [file 1471-2172-6-17-S1.pdf]

Protocol for Isolation, Cryopreservation, and Thawing of PBMCs

Description

Cryopreserved PBMCs are a common specimen source for studies of immunological responses to vaccines, immunotherapies, etc. The health and viability of cells recovered post-cryopreservation are of course critical to the success and accuracy of immunological assays performed on them. We have developed this protocol to help standardize PBMC isolation and cryopreservation techniques, specifically for the assessment of thawed cells by cytokine flow cytometry.

Materials and Methods

Table 1 Reagents

Reagents	Source	Catalog Number
DMSO	Sigma	D2650
RPMI-1640, sterile, L-glutamine and HEPES supplemented	Sigma	R7388
Antibiotic/antimycotic solution	Sigma	A9909
Fetal bovine serum (FBS)	Sigma	F2442
Trypan blue, 0.4% solution	Sigma	T8154
Human albumin fraction V, low endotoxin	Gemini Bioproducts	800-120

Table 2 Accessory Products and Instrumentation

Product	Source	Catalog Number
BD Vacutainer™ CPT™ Tube with sodium heparin, 8 mL	BD Biosciences, Discovery Labware	362753
Disposable polystyrene serological pipette: 5 mL<br>10 mL	BD Biosciences, Discovery Labware	357543<br>357551
Sterile polypropylene conical tube, 50 mL	BD Biosciences, Discovery Labware	352070
1.8 and/or 3.6 ml cryovial, Nunc or equivalent[a]		
96-well round bottom plate with lid	BD Biosciences, Discovery Labware	353077
Hemacytometer (Fisher or equivalent)		
200 μl micropipettor (Pipetman or equivalent) with sterile tips		
Serological pipettor (Pipet-Aid or equivalent)		
Freezing containers, Nalgene "Mr. Frosty" or equivalent		
Microscope (Photozoom, Nikon, Zeiss, or equivalent)		
37°C water bath		
37°C and 5% CO_2 incubator		
Refrigerated table top centrifuge (e.g. Sorvall RT6000 centrifuge)		
Biological safety cabinet, Class II (NuAire, Baker, or equivalent)		

Please follow all recommended precautions that are provided in the technical data sheet of each manufacturer's product.

Instructions for Processing Reagents

Complete RPMI (cRPMI)

Supplement sterile RPMI-1640 medium with 10% sterile heat-inactivated FBS and 1% sterile antibiotic/antimycotic. Store at 4°C.

Stock 25% Human Serum Albumin (HSA)

Prepare 25% HSA in RPMI; e.g. dissolve 25 gm human albumin fraction V into 100 ml RPMI (not complete RPMI). Allow 24 hrs to fully dissolve. Store at 4°C.

12.5% Human Serum Albumin

Combine 10 ml of stock 25% HSA and 10 ml of sterile RPMI-1640 medium (not complete RPMI). Store at 4°C.

2X Freezing Medium

Combine 10 ml of stock 25% human serum albumin and 10 ml of sterile RPMI-1640 medium (not complete RPMI). Add 5 ml of DMSO. Store at 4°C.

Processing of Fresh PBMCs

Isolation

1. Collect blood via venipuncture directly into CPT tube(s).
2. Store CPT tube(s) at room temperature if they cannot be processed immediately; cell degradation will occur if tubes are stored for more than four hours.
3. Centrifuge CPT tube(s) at 1800 x g (approximately 2800 rpm on a Sorvall RT6000 centrifuge) for 20 minutes at room temperature. Be sure that the tubes are not loaded in the outer positions of the carriers, as this may cause the rubber tops of the tubes to contact the rotor and come off.
4. After centrifugation, bring the CPT tube(s) to a biological safety cabinet and carefully open the tops. Using a 5 ml pipette, gently pipette the plasma up and down against the gel plug to dislodge cells that are stuck to the top of the gel. Avoid vigorous pipetting that would disintegrate the gel plug itself.
5. Transfer the cell suspension from the CPT tube(s) to a 50 ml conical polypropylene tube, pooling the cells from each tube if there are multiple CPT tubes per donor. Add cRPMI to a total of 40 ml.
6. Remove a 10 μl aliquot of cell suspension for counting as per **Cell Counting With a Hemacytometer, and Determining Cell Viability With Trypan Blue** section, following.
7. Centrifuge 50 ml tubes at 250 x g (approximately 1200 rpm on a Sorvall RT6000 centrifuge) for seven minutes at room temperature.
8. Count cells with hemacytometer while tubes are centrifuging.
9. When centrifugation is complete, aspirate the supernatant and gently flick the tube with a finger to break up the pellet.
10. a) If cells are to be used fresh: Resuspend the cells at 5 x 10^6 viable lymphocytes per ml in cRPMI. Proceed with functional assay as per protocol..
    b) If PBMCs are to be cryopreserved: Prepare reagents for cryopreservation in advance. If this is not possible, make sure the cell pellet is disrupted and store the cells on ice until all reagents are prepared. Resuspend the cells as per **Cryopreservation of PBMCs** section, following.

Cell Counting With a Hemacytometer, and Determining Cell Viability With Trypan Blue

Note: Trypan blue is one of several stains recommended for use in dye exclusion procedures for viable cell counting. This method is based on the principle that live (viable) cells will not take up certain dyes, whereas dead (non-viable) cells will.

1. Mix 10 µl of cell suspension with 10 µl of 0.4% trypan blue. Allow dilution to incubate for three to five minutes at room temperature.
2. Inject 10 µl of the trypan blue/cell mixture beneath the cover slip on a hemacytometer. This volume will ensure that the hemacytometer is not overfilled. Place the hemacytometer on the stage of a binocular microscope and focus on the cells.
3. Count the unstained (viable) and stained (non-viable) cells from the central large square of the hemacytometer. This square should just fill the field of view when using the 10X lens. If the total cell count is less than 50, count additional large squares until between 50 and 100 cells have been counted.
4. Calculate the total number of viable cells as follows:

   Total # viable cells = # viable cells per square x 2 x 10,000 x total volume cell suspension (in ml)

5. Calculate the percentage of viable cells as follows:

$$\%\ \text{viable cells} = \frac{\#\ \text{viable cells counted}}{\#\ \text{total cells counted}} \times 100$$

6. Rinse the hemacytometer and cover slide with 70% alcohol, and wipe dry.

Cryopreservation of PBMCs

The following protocol for freezing PBMCs uses a final concentration of 10% dimethylsulfoxide (DMSO) and 11.25% protein (human serum albumin) in cRPMI. Cryoprotectants, such as DMSO, reduce the amount of ice present during freezing and reduce solute concentration, thus reducing ionic stress. However, these compounds can themselves cause osmotic injury since they are hypertonic and can cause damage during their addition or removal.

1. Resuspend PBMCs (from **Isolation** section of **Processing of Fresh PBMCs**, above) at 1 x 10^7 viable lymphocytes/ml in 4°C 12.5% HSA in RPMI medium, in a 50 ml conical polypropylene tube.
2. While *gently* swirling the tube, add dropwise enough 4°C 2X freezing medium to double the volume of the cell suspension.
3. Immediately place the tube on ice. Avoid any further mixing or agitation of the cells. Slowly remove the cell suspension into a pipet and dispense 1 ml per cryovial on ice.
4. Place the cryovials in a pre-cooled Mr. Frosty-style freezing container that has been filled with 70% isopropanol according to the manufacturer's instructions. Place the freezing container at –80°C.

Thawing of PBMCs

If PBMCs are not thawed properly, viability and cell recovery can be compromised; and cells may not perform optimally in functional assays. In general, cells should be thawed quickly but diluted slowly to remove DMSO. Cells with DMSO intercalated into their membranes are very fragile, and must be pelleted and handled gently.

1. Warm cRPMI to 22°-37°C in a 37°C water bath before beginning thawing procedure.
2. Transfer the cryovial from liquid nitrogen to a 37°C water bath. If liquid nitrogen has seeped into the cryovial, loosen the cap slightly to allow the nitrogen to escape during thawing.

3. Hold the cryovial in the surface of the water bath with an occasional gentle “flick” during thawing. Do not leave the cryovial unattended during the thawing process. It is important for cell viability that the cells are thawed and processed quickly; thawing takes only a minute or two. When a small bit of ice remains in the cryovial, transfer the cryovial to the biosafety hood. Dry off the outside of the cryovial and wipe with disinfectant before opening to prevent contamination.
4. Add warm cRPMI dropwise into the cryovial containing the cell suspension, slowly over a 30 second period. The final volume should be twice the volume of the cell suspension (e.g., add 1 ml cRPMI to a cryovial containing 1 ml cell suspension). Be careful not to exceed the capacity of the cryovial.
5. Transfer the diluted cell suspension to a 50 ml polypropylene centrifuge tube containing 8 ml of warm cRPMI for every vial of cells added (multiple cryovials from the same donor may be combined into one 50 ml tube, if desired).
6. Centrifuge the cells at 1200 rpm for seven minutes. Decant the supernatant, and gently flick the tube with a finger to break up the pellet. Resuspend in desired volume of warm cRPMI.
7. Determine cell number and viability as per **Cell Counting With a Hemacytometer, and Determining Cell Viability With Trypan Blue** section, above.
8. If cells require concentrating, centrifuge at 1200 rpm for seven minutes. Decant the supernatant, and gently flick the tube with a finger to break up the pellet.
9. Dilute to a final working concentration of $5x10^6$ PBMCs/mL in room temperature cRPMI media.
10. Check for clumps and remove them with a pipettor tip.
11. For cytokine assays, plate 200 μl/well in a round-bottom 96-well plate. This will result in $1x10^6$ cells per well (we have tested $5x10^5$ to $2x10^6$ cells per well with equivalent results). Prepare additional wells for manual compensation, if desired.
12. Incubate covered plate at 37°C for 12–18 hours to rest cells. Proceed with functional assay as per protocol.
